# Plant-Parasitic Nematodes Associated with Strawberry and Molecular Identification of *Meloidogyne* and *Pratylenchus* Species in the Central Region of Costa Rica

**DOI:** 10.2478/jofnem-2025-0033

**Published:** 2025-08-31

**Authors:** Ricardo Brenes-Campos, Lester A. Núñez-Rodríguez, Lorena Flores-Chaves, Danny A. Humphreys-Pereira

**Affiliations:** Laboratory of Nematology, Crop Protection Research Center (CIPROC), Agronomy School, University of Costa Rica, San José, 2060, Costa Rica

**Keywords:** *COI*, *Meloidogyne*, molecular identification, nematodes, *Pratylenchus*, strawberry

## Abstract

Strawberries are primarily cultivated in the Central Region of Costa Rica due to the favorable growing conditions there. However, several factors can affect the final yield and quality of strawberries, including the presence of plant-parasitic nematodes (PPN). Unfortunately, no surveys have been conducted in the country to identify the PPN affecting production. This study aimed to identify morphologically PPN genera associated with strawberry in the Central Region of Costa Rica, and to identify the *Meloidogyne* and *Pratylenchus* species using molecular techniques. A nematode survey was performed between 2018 and 2021 across four provinces: Cartago, Alajuela, Heredia, and San José. The most frequent nematodes found in both root samples (n = 55) and soil samples (n = 53) were *Meloidogyne* (Frequency of occurrence, FO = 78% in root and 62% in soil) and *Pratylenchus* (FO = 56% and 43%, respectively) (*p* < 0.05). Molecular techniques with species-specific primers, such as PCR-RFLP and PCR, allowed for the identification of 13 *Meloidogyne* populations, all confirmed to be *M. hapla*. DNA sequencing of the partial mitochondrial *COI* gene and PCR with species-specific primers found 11 *Pratylenchus* populations, with 10 identified as *P. penetrans* and one as *P. hippeastri*. Further studies should focus on pathogenicity assays with a diversity of strawberry cultivars to assess damage potential and develop strategies for integrated management of PPN in strawberry production.

In Costa Rica, strawberry production is concentrated in areas with an altitude between 1,300 to 2,000 m a.s.l. with temperate conditions (MAG, 2017; Abdallah, 2015). These conditions predominate in some areas of the Central Region. Most of the production is in the provinces of Cartago, Alajuela, Heredia, and San José (INEC, 2015; SEPSA, 2018). Strawberry cultivars grown in Costa Rica include ‘Chandler’, ‘Oso Grande’, ‘Camino Real’, ‘Festival’, ‘Elyana’, ‘San Andreas’, and ‘Albion’; the last two are the most popular among farmers (MAG, 2017).

The most common system for strawberry cultivation in Costa Rica is the use of raised beds covered with plastic mulch, either in the greenhouse or in an open field. This traditional production system may be contributing to infection by soil-borne pathogens such as the fungi *Fusarium oxysporum*, *Neonectria*/*Dactylonectria* and *Sydowia polyspora* (Granados-Montero et al., 2022). Between 2016 and 2017, strawberry production in Cartago declined by 93 ha, and this was attributed to a fungal disease complex in soil that caused plant stunting and decline (Granados-Montero et al., 2022). PPNs have reportedly caused yield losses of approximately 12% in strawberries in other countries (Abd-Elgawad, 2019), though this number may increase due to interaction between soil-borne pathogens and PPNs (LaMondia, 1999).

There is a lack of information about the PPNs associated with strawberries in Costa Rica. The identification of PPNs at the species level may allow for the application of integrated management approaches, such as crop rotation, soil improvement, and introduction of natural enemies of nematodes such as nematophagous fungi or parasitic bacteria, along with other ecological methods (Feyisa, 2022). The most harmful PPNs on strawberry are *Meloidogyne hapla*, *Pratylenchus penetrans*, *Aphelenchoides fragariae*, *A. besseyi*, and *Belonolaimus longicaudatus*; the last one is a serious pest only in Florida (Nyoike et al., 2012; Oliveira et al. 2019; SAG, 2022; Watson et al., 2020).

Currently, some of the most widely used molecular techniques for the identification of PPNs are PCR with species-specific primers, PCR-RFLP, and sequencing (Powers and Harris, 1993; Janssen et al., 2017). In *Meloidogyne*, the mitochondrial DNA region between the genes cytochrome oxidase subunit II (COII) and 16S rRNA has been used in combination with RFLP to identify species (Powers and Harris, 1993). The most common DNA markers for the identification of *Pratylenchus* species include the partial mitochondrial cytochrome oxidase subunit I (COI) gene and ribosomal genes such as the 18S, ITS, and 28S (Subbotin et al., 2008; De Luca et al., 2011; Janssen et al., 2017).

Some strawberry cultivars used in Costa Rica, such as ‘Oso Grande’, ‘Albion’, ‘Camino Real’, ‘Festival’, and ‘San Andreas’, have been shown to be resistant to *M. incognita, M. javanica*, and *M. hapla*, as well as *P. zeae* and *P. brachyurus,* in greenhouse and field experiments in Brazil (Curi et al., 2016; Brum et al., 2019). In Costa Rica, surveys of the PPNs associated with strawberry have not been conducted, but *M. hapla* and *P. penetrans* have been reported in a few strawberry farms (Humphreys et al., 2012; Sandoval et al., 2023). To promote specific management practices in strawberry production fields — such as the use of resistant cultivars, crop rotation, and biological/chemical control — it is necessary to know the most important PPN species in the main productive areas of the country. The objectives of this study were thus to (i) determine the frequency and distribution of the genera of PPNs associated with strawberry in the Central Region of Costa Rica, and (ii) identify the *Meloidogyne* and *Pratylenchus* species using molecular techniques.

## Materials and Methods

### Sampling and nematode populations

A survey of PPNs was conducted from 2018 through 2021 in the Central Region of Costa Rica. Twenty-six farms were included in the study. Each farm often consisted of more than one field. The survey covered the provinces of Cartago (10 farms, representing 18 fields); Alajuela (6 farms, 16 fields); Heredia (7 farms, 16 fields); and San José (3 farms, 5 fields). In total, 53 root, soil and foliage composite samples were collected. In one farm in Heredia, the farmer collected two composite root samples and submitted them to the Laboratory of Nematology (CIPROC) at the Agronomy School at the Universidad de Costa Rica, San Pedro, San José; this increases the number of composite root samples to 55. In this study, only fields that had been established for at least two months were considered, so that they would have enough root material for sampling. Though the presence or absence of nematode damage symptoms did not serve as a selection criterion, it was observed that some fields exhibited symptoms of stunting, whereas others did not. Within each field, a composite tissue or soil sample was collected exclusively from plants that were all of the same variety and age. Each composite sample consisted of five entire plants (root system, crown and foliage) and the soil surrounding the roots (~2 kg). These plants and soil were collected in a zig-zag pattern and placed in a bag.

Nematode extraction was carried out as described by Sandoval-Ruíz et al., (2023)s. Briefly, roots and foliage were washed separately, cut into 1-3-cm-long pieces, and a subsample of 10 g of each tissue was processed separately by the flotation-centrifugation method (Caveness and Jensen, 1955; Alvarado and López, 1985). For soil samples, nematodes were extracted from a subsample of 100 g of soil using the same flotation-centrifugation method. Using an inverted light microscope (Nikon TS100, Japan), all PPNs found in the samples were identified to genus based on morphology.

The frequency of occurrence (FO%) of each genus was calculated as in Barker (1985): FO% based on the total number of samples (number of samples where a nematode genus was present, divided by total number of samples) × 100 and FO% accounting for province (number of samples where a nematode genus was present, divided by number of samples per province) × 100. Maximum and mean population densities were also estimated in both cases. The frequency of occurrence was analyzed by the χ^2^ test for independence for the effects of province. Kruskal-Wallis and Mann-Whitney tests were used to analyze the effect of province on PPN population densities. Also, the relative frequency (RF) was calculated only for the three nematodes with the highest FO % in roots (Barker, 1985) and their possible combinations with each other.

### Molecular identification of *Meloidogyne*

Thirteen field populations of *Meloidogyne* were randomly selected for molecular identification from four provinces (Table 1). In this study, a population is defined as a group of individuals extracted from a single root composite sample collected from a specific farm. For each population, eight DNA samples (each consisting of five second-stage juveniles) were prepared as in Sandoval-Ruiz et al. (2020). The mitochondrial region between the COII and the 16S rRNA genes was amplified using the primers C2F3 (5′-GGTCAATGTTCAGAAATTTGTGG-3′) and 1108 (5′-TACCTTTGACCAATCACGCT-3′) (Powers and Harris, 1993).

**Table 1:** Populations of *Meloidogyne* spp. and *Pratylenchus* spp. collected from strawberry fields, including information on variety and localities, in the Central Region of Costa Rica.

**Population code^a^**	**Variety**	**Locality**
M1	Albion	Llano Grande, Cartago, Cartago
M2	San Andreas	Llano Grande, Cartago, Cartago
M3	Albion	Llano Grande, Cartago, Cartago
M4	Festival	Llano Grande, Cartago, Cartago
M5	San Andreas	San Nicolás, Cartago, Cartago
M6	Albion	San Nicolás, Cartago, Cartago
M7	San Andreas	Varablanca, Varablanca, Heredia
M8	Albion	Varablanca, Varablanca, Heredia
M9	Albion	Varablanca, Varablanca, Heredia
M11	Elyana	Sabanilla, Alajuela, Alajuela
M12	San Andreas	Sabanilla, Alajuela, Alajuela
M13	San Andreas	Copey, Dota, San José
P1	Albion	Llano Grande, Cartago, Cartago
P2	San Andreas	Llano Grande, Cartago, Cartago
P3	Festival	Llano Grande, Cartago, Cartago
P4	Camino Real	Llano Grande, Cartago, Cartago
P5	San Andreas	Llano Grande, Cartago, Cartago
P6	Elyana	Sabanilla, Alajuela, Alajuela
P7	San Andreas	Sabanilla, Alajuela, Alajuela
P8	Albion	Sabanilla, Alajuela, Alajuela
P9	Elyana	Sabanilla, Alajuela, Alajuela
P10	San Andreas	Copey, Dota, San José
P hip^b^	San Andreas	Varablanca, Varablanca, Heredia

a M= *Meloidogyne*, P= *Pratylenchus*.

b Previously identified as *P. hippeastri* by Brenes-Campos et al. (2021).

The amplification reaction included 1 × DreamTaq PCR Buffer (Thermo Fisher Scientific, USA), 0.6 μM of each primer, 1 μl of 20 mg/ml BSA (Thermo Fisher Scientific, Lithuania), 0.1 mM dNTP mix (Thermo Fisher Scientific, Lithuania), 1.5 units of DreamTaq polymerase (Thermo Fisher Scientific, USA), 3 μl of the DNA preparation in a final volume of 25 μl. The PCR amplification was performed in a Mastercycler Pro thermal cycler (Eppendorf, Germany). The amplification conditions described by Jeyaprakash et al. (2006) were followed. DNA amplification products were visualized by electrophoresis using a 1% agarose gel (1 g of agarose on 100 ml of 0.5 × TBE) mixed with DNA Gel Loading Dye 6X (Thermo Fisher Scientific, USA) and GelRed (Biotium, USA) following the manufacturers’ instructions. Electrophoresis was run at 110 V for 1 h and observed in a BioDoc-It^2^ 315 Imaging System LMS-26 (UVP, USA) transilluminator.

PCR products were digested with the restriction enzyme DraI (Thermo Fisher Scientific, USA) according to the manufacturer’s instruction. The restriction patterns were visualized by electrophoresis using a 2% agarose gel. Species identification was confirmed with a multiplex PCR using the species-specific primers JMV1 (5′-GGATGGCGTGCTTTCAAC-3′), JMV2 (5′-TTTCCCCTTATGATGTTTACCC-3′), and JMVhapla (5′-AAAAATCCCCTCGAAAAATCCACC-3′) (Wishart et al., 2002). A positive control of *M. hapla* collected from golden berry (*Physalis peruviana*) in Llano Grande, Cartago was included in the molecular assays.

### Molecular identification of *Pratylenchus*

Ten populations of *Pratylenchus* were randomly selected (Table 1), identified through COI sequencing, and confirmed with species-specific primers. Nematode cultures were established using 25 females from each population and cultured on carrot discs at 28° C (Coyne et al., 2014) for three months. Pure nematode cultures were used for further molecular analysis. DNA was extracted from ~1,000 nematodes using the GeneJET Genomic DNA Purification Kit (Thermo Fisher Scientific, USA) following the manufacturer’s instructions. Four combinations of primers were used for the *COI* amplification: JB3 (5′-TTTTTTGGGCATCCTGAGGTTTAT-3′)/JB4.5 (5′-TAAAGAAAGAACATAATGAAAATG-3′), JB3/JB5 (5′-AGCACCTAAACTTAAAACATAATGAAAATG-3′) (Bowles et al., 1992; Derycke et al., 2005), F7bP (5′-GGDTGRACWTTH TAYCCNCC-3′)/JB4.5 or F7bP/JB5 (Ozbayrak et al., 2019). The amplification reaction included 1× DreamTaq PCR Buffer (Thermo Fisher Scientific, USA), 0.8 μM of each primer, 1 μl of 20 mg/ml BSA (Thermo Fisher Scientific, Lithuania), 0.1 mM dNTP mix (Thermo Fisher Scientific, Lithuania), 1.5 units of DreamTaq polymerase (Thermo Fisher Scientific, USA), 3 μl of the DNA preparation in a final volume of 25 μl. The amplification conditions described by Abarca-Durán et al. (2022) were followed. PCR products were cleaned up and sequenced bidirectionally at Macrogen (Seoul, South Korea). At least eight COI sequences were obtained from each population.

Sequences generated in this study were aligned using ClustalW (Thompson et al., 1994) in Bioedit v.7.0.5.3 (Hall, 1999) with sequences retrieved from GenBank. Phylogenetic relationships within the genus *Pratylenchus* were estimated with the Bayesian Inference method in MrBayes v.3.2 (Ronquist et al., 2012). The substitution model GTR + I + G was selected as the best-fit nucleotide model using the program JmodelTest v2.1.10 (Darriba et al., 2012). The Bayesian analysis was conducted with four MCMC chains (three heated, one cold) and 106 generations (sampled every 1,000 generations). The first 2,500,000 generations (2,500 trees) were discarded as burn-in. The tree topologies were visualized using the program FigTree v. 1.4.3 (Rambaut, 2012).

The identity of *P. penetrans* was confirmed with species-specific *β*-1,4-endoglucanase gene primers PP5F (5′-ACATGGTCGACACGGTGATA-3′) and PP5R (5′-TGTTGCGCAAAT CCTGTTTA-3′) (Mekete et al., 2011). PCR was carried out according to the author’s recommendation. The amplification products were visualized as described above.

### Molecular identification of *Aphelenchoides*

Presence of *Aphelenchoides* was detected and identified at the species level in only one of the strawberry foliar samples. The COI was amplified and sequenced in this population. DNA was extracted from one individual nematode (n = 8) using the GeneJET Genomic DNA Purification Kit (Thermo Fisher Scientific, USA) following the manufacturer’s instructions. The primers COI-F1 (5′-CCTACTATGATTGGTGGTTTTGGTAATTG-3′) and COI-R2 (5′-GTAGCAGCAGTAAAATAAGCACG-3′) (Kanzaki and Futai, 2002) were used for amplification. PCR conditions and visualization methods were the same as the ones described above for *Pratylenchus*.

## Results

### Frequency and population density of PPNs

Thirteen different PPNs (10 genera and three families) were found associated with strawberry (among root and soil samples) in the Central region of Costa Rica (Table 2). At least one genus of PPN was found in 94.5% of the total number of root samples, and 92.4% of the soil samples. In root and soil samples, the most frequent PPNs associated with strawberry were *Meloidogyne* (present in 78% of root and 62% of soil samples) and *Pratylenchus* (56% of root and 43% of soil samples) (*P* < 0.05) (Table 2).

**Table 2:** Frequency of occurrence (FO%), minimum (Min), maximum (Max), and mean population density of plant-parasitic nematodes when present in samples of root (nematodes/100 g of roots) or soil (nematodes/100 g of soil) collected from strawberry fields in the Central Region of Costa Rica.

**Genus/family**	**Root^a^**			**Soil^b^**		

	**FO%^c^**	**Mean**	**Min - Max**	**FO%^c^**	**Mean**	**Min - Max**
*Meloidogyne*	78a	20,407	10 - 265,600	62a	1,536	1 - 26,800
*Pratylenchus*	56a	4,853	10 - 33,520	43a	453	1 - 9,600
*Helicotylenchus*	25b	134	10 - 420	30b	23	1 - 120
Criconematidae	16b	740	10 - 5,120	32b	240	1 - 1,560
*Hemicycliophora*	15b	619	10 - 3,020	28b	362	1 - 2,160
Heteroderinae J2	11b	12	10 - 20	2b	10	10 - 10
*Aphelenchoides*	2b	15	15 - 15	6b	2	1 - 3
Trichodoridae	-^d^	-	-	4b	27	50 - 50
*Rotylenchulus*	2b	20	20 - 20	-	-	-
*Rotylenchus*	-	-	-	2b	1	1 - 1
*Ditylenchus*	-	-	-	2b	1	1 - 1
*Xiphinema*	-	-	-	2b	1	1 - 1
*Gracilacus*	-	-	-	2b	20	20 - 20

a Number of samples included in the analysis was 55.

b Number of samples included in the analysis was 53.

c Frequency of occurrence data were analyzed for effects using χ^2^ analysis. Values followed by the same letter in the same column are not significantly different from each other (*P* < 0.05).

d Not present.

The most abundant PPNs in roots and soil (highest average number of nematodes, when present, per 100 g of roots or 100 g of soil) were *Meloidogyne* and *Pratylenchus* (Table 2). In roots, the average population density of *Meloidogyne* was 20,407 second-stage juveniles (J2) (ranging from 10 to 265,600) per 100 g of root, while that of *Pratylenchus* was 4,853 nematodes per 100 g of root (10 to 33,520). In soil samples, the population densities of *Meloidogyne* and *Pratylenchus* averaged 1,536 J2 per 100 g of soil (ranging from 10 to 26,800), and 453 nematodes per 100 g soil (ranging from 1 to 9,600), respectively. No statistical differences were found between PPN genera densities in roots and soil (data not shown).

The interaction between the three most frequent PPN genera (*Meloidogyne*, *Pratylenchus*, and *Helicotylenchus*) in roots was analyzed by the relative frequency (no significant differences were found). It was observed that *Meloidogyne* and *Pratylenchus* (MP) were found together in only 31% of the samples. *Meloidogyne* appeared alone in 25%; the combination of *Meloidogyne*, *Pratylenchus* and *Helicotylenchus* (MPH) in 15%; *Meloidogyne* and *Helicotylenchus* (MH) in 7%; *Pratylenchus* alone in 7%; and *Pratylenchus* and *Helicotylenchus* (PH) in 4%. Samples with none of these three genera accounted for 11%.

No symptoms associated with foliar nematodes were observed in the fields. However, *Aphelenchoides* was detected in one composite foliage sample collected in the locality of Fraijanes, Alajuela. This sample had 114 *Aphelenchoides* per 100 g of foliage. Eight individual DNA samples were used for PCR amplification of the COI. Successful amplification, obtained from only two samples, was sequenced bidirectionally. The two sequences were identical and had a 682-bp length (GenBank accession number OR462349). The sequence had 88.5% similarity with an *Aphelenchoides* sp. sequence recovered from *Pinus radiata* (EU287593); 87.5% with *A. paradalianensis* obtained from *Pinus tabuliformis* (MT808399); and 87.4% with *A. xylocopae* (cultured on *Botrytis*).

### Molecular identification of *Meloidogyne*

PCR amplification of the mitochondrial region between the COII and 16S rRNA genes of 13 *Meloidogyne* populations (Table 1) extracted from strawberry root samples and a *M. hapla* positive control yielded a single band of ~500 bp. Digested PCR products showed one restriction pattern of two bands (~200 bp and ~250 bp) in all samples, including the positive control. PCR using species-specific primers (JMV1, JMV2 and JMVhapla) for *M. hapla* yielded a single band of ~440 bp in all samples (data not shown).

### Molecular identification of *Pratylenchus*

The analysis of 99 *Pratylenchus* COI sequences (trimmed to a length range of 393 to 402 bp) from 10 populations revealed four *haplotypes* based on 19 DNA substitutions. All of them were synonymous mutations. Within each population, only one haplotype was present. Thus, only the four haplotype sequences were used for further analyses. There was 99.7 to 100% similarity between the sequences obtained in this study and sequences retrieved from GenBank for *P. penetrans* (Table 3). Additionally, in this survey an unreported *Pratylenchus* species from the province of Heredia was described as *P. hippeastri*, with identity percentages of 100% for COI and ITS markers, and 98% for 28S (Brenes-Campos et al., 2021).

**Table 3:** Similarity of the *Pratylenchus* sequences generated in this study, with the GenBank accessions based on the partial *COI* gene and number of sequences per haplotype on each population.

**Population code (GenBank accession^a^)**	**Number of sequences**	**Collection area**	**Haplotype**	***Pratylenchus* species**	**Identity (%)**	**Matched accession from GenBank**
P1 (PP550689)	13	Cartago	PPH1	*P. penetrans*	100	MT527068
P6 (PP550690)	10	Alajuela				KY816944
						MW660615
P2 (PP550684)	8	Cartago	PPH2	*P. penetrans*	100	MN445196
P3 (PP550688)	10					MK877997
P4 (PP550687)	10					KY816936
P5 (PP550691)	11					
P7 (PP550685)	8	Alajuela				
P10 (PP550686)	9	San José				
P8 (PP550683)	11	Alajuela	PPH3	*P. penetrans*	100	KY816948
					99.75	KY816961
					99.75	MW660615
P9 (PP544792)	9	Alajuela	PPH4	*P. penetrans*	100	MT527043
					99.75	KY816960
					99.75	MT527066
P hip^b^	11	Heredia	PHH1	*P. penetrans*	97	KY424098, KY424099
(MW680342)						MW042870

a Sequences from this study.

b Previously identified as *P. hippeastri* by Brenes-Campos et al. (2021).

**Figure 1:**
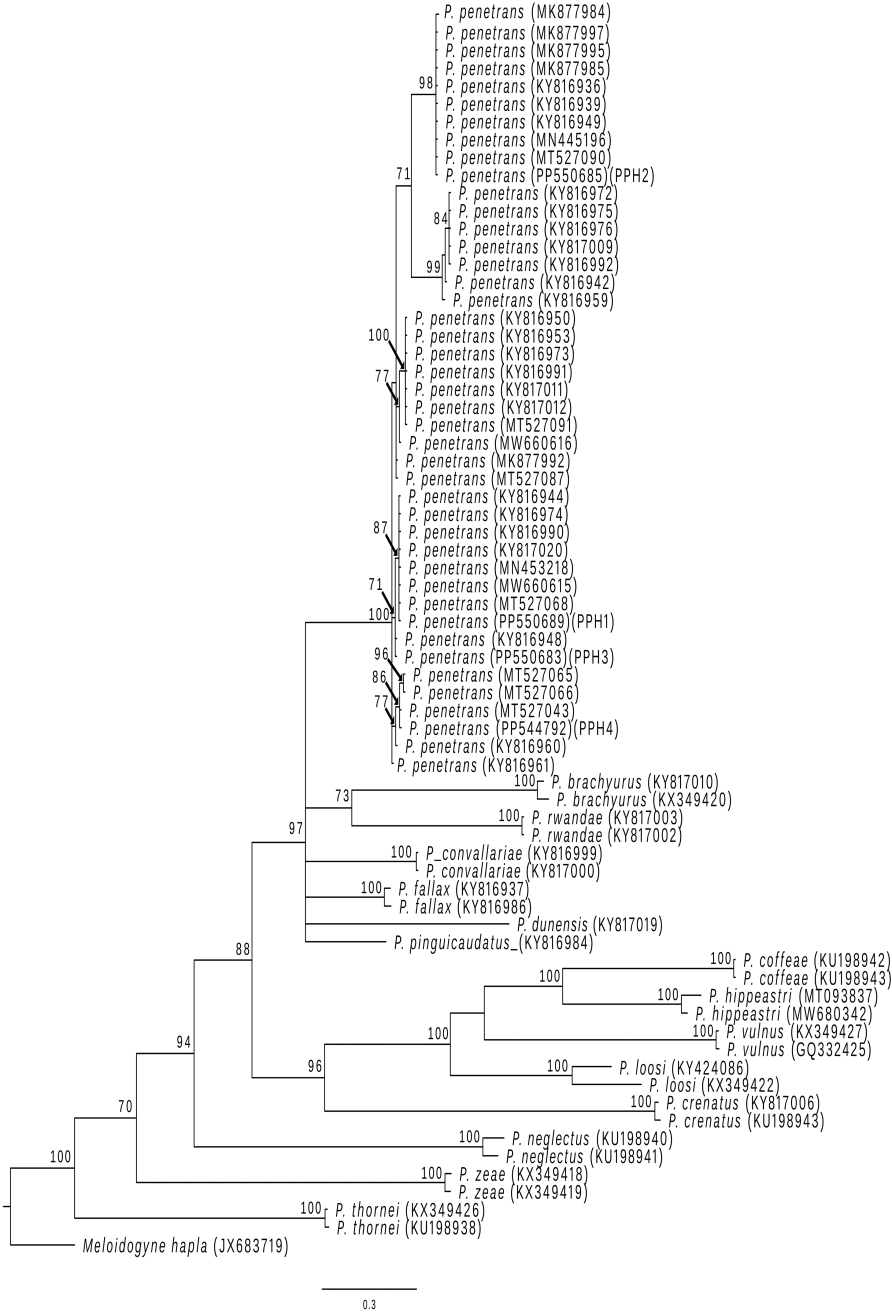
Phylogenetic relationships between *Pratylenchus* species as inferred from Bayesian analysis of the COI gene sequences using the GTR + I + G model of nucleotide substitution. Posterior probabilities of over 70% are given for appropriate clades. Sequences generated in this study show the haplotype in parenthesis (PPH1, PPH2, PPH3 and PPH4).

The phylogenetic relationships based on the COI showed that the four haplotype sequences generated in this study were placed in a large monophyletic group denominated as the *Penetrans* species complex (Bayesian Posterior Probability, BPP = 97%) (Janssen et al., 2017). Within this clade, all four Costa Rican COI haplotypes were placed in a subclade that conformed only with *P. penetrans* sequences from different countries and hosts (BPP = 100%). Each Costa Rican COI haplotype was positioned on a different group, but only the one with the PPH2 haplotype showed a high BPP value, at 98% (Fig. 1). This haplotype was the most prevalent, appearing in six out of 10 populations of *P. penetrans* extracted from strawberries, and was distributed across three of the four provinces in the Central Region.

All identifications of Costa Rican populations as *P. penetrans* by sequencing were also corroborated using the PCR-species specific primers PP5F/PP5R. The results showed a single band of ~500 bp in all *Pratylenchus* populations (data not shown).

## Discussion

This survey provides valuable information on the occurrence and population densities of PPNs associated with strawberry in the Central Region of Costa Rica. A total of 10 genera and members of three families of PPNs were detected in this region. This great diversity of PPNs coincides with other nematological studies conducted in strawberries in Spain and Brazil (Talavera et al., 2019; Krezanoski et al., 2020). In Costa Rica, the most frequent PPNs associated with the crop were *Meloidogyne* and *Pratylenchus*, which are also recognized as the most important PPNs in strawberry production worldwide (Esnard and Zuckerman, 1998). Significantly, the foliar nematode *A. fragariae* was not found in this study, and the dagger nematode was only found at a very low density in one field in the province of San José.

Despite the high sand-particle content (50%–90%, data not shown) in some strawberry planting areas in the country, the sting nematode *Belonolaimus longicaudatus* was not found in this study. This species is known to cause severe damage to strawberries in Florida (Watson et al., 2020). In Costa Rica, *B. longicaudatus* has only been identified in the Pacific coast in low and warm areas (López, 1979), where strawberries are not cultivated.

In this survey, the genus *Meloidogyne* was the most prevalent PPN found in both root and soil samples, and had the highest average population density. The abundance and frequent occurrence of *Meloidogyne* spp. in strawberries has also been reported in other strawberry-producing countries, such as Spain (Talavaera et al., 2019). In contrast, *Helicotylenchus* was the most frequent and abundant nematode found in Paraná, Brazil (Krezanoski et al., 2020). *Pratylenchus* was the second most frequent PPN found in this survey. This trend has also been observed in other countries — such as Spain, Brazil, and Canada — where *P. penetrans* has been reported as the second-most prevalent PPN in soil samples (Atterson 2015; Talavaera et al., 2019; Krezanoski et al., 2020). Variations in nematode distribution could be linked to soil chemical factors, management practices, environmental conditions, and the tolerance of strawberry cultivars to nematodes (Edwards et al., 1895).

The molecular techniques allowed for the identification of 13 *Meloidogyne* populations as *M. hapla.* This species has been previously reported in strawberry fields in the Central Region and is widely distributed in temperate regions of Costa Rica (López and Salas, 1978; Humphreys et al., 2012). Therefore, the presence and dominance of *M. hapla* over other species found in this survey of strawberry fields is to be expected, and has been reported by Bélair and Khanizadeh (1994). Although *M. hapla* was the only root-knot nematode species identified in this study, other species have been reported in strawberry, including *M. incognita, M. arenaria,* and *M. javanica,* with *M. hapla* appearing in the USA, Spain, and Brazil (Nyoike et al., 2012; Talavera et al., 2019; Krezanoski et al., 2020). *M. hapla* has been reported to be the predominant *Meloidogyne* species in strawberry fields in Canada, USA, Spain, and Brazil (Bélair and Khanizadeh, 1994; Nyoike et al., 2012; Talavera et al., 2019; Krezanoski, et al., 2020). In southern Spain, the frequency of occurrence of *M. hapla* was 71%, compared with 8% for *M. incognita* and 6% for *M. javanica* (Talavera et al., 2019).

Curi et al. (2016) reported that the strawberry cultivar ‘Albion’ is resistant to *M. hapla* under Brazilian conditions, while the cultivar ‘Festival’ was classified as susceptible. Brum et al. (2019) confirmed the resistance of the cultivar ‘Albion’, they reported contrasting results, demonstrating resistance in both cultivars ‘Festival’ and ‘San Andreas’. The results suggest that resistance levels may differ depending on specific environmental conditions, and that local factors, such as soil properties and crop-management practices, might be influencing the nematode-plant interaction.

The study of the COI allowed for a separation of the Costa Rican populations of *P. penetrans* from all the other species belonging to the *Penetrans* group (including *P. penetrans*, *P. fallax*, *P. convallariae*, *P. pinguicaudatus*, *P. dunensis*, *P. rwandae,* and *P. oleae*) (Janssen et al., 2017; Singh et al., 2018). In this group, morphometric characteristics are not useful for species diagnosis because of the large intraspecific morphological variability (Janssen et al., 2017).

The identified haplotypes are present in *P. penetrans* populations worldwide. In Costa Rica, the PPH2 haplotype was found to be the most prevalent in strawberry. This haplotype has also been reported in the Netherlands and the USA, in crops such as apple, cherry, potato, corn and peony (Janssen et al., 2017; Ozbayrak et al., 2019; Cole et al., 2020). The presence of a single haplotype within a population may indicate an introduction from a particular country to a specific strawberry-producing region of Costa Rica. The introduction of strawberry planting materials from the USA, Mexico, Chile, Argentina, Spain, and the Netherlands has been documented by the Ministry of Agriculture. Although *P. penetrans* was the most common species found in this study, species such as *P. vulnus*, *P. crenatus, P. neglectus*, and *P. pratensis* have been reported in mixed populations in strawberry fields across the world (Yu, 2008; Kochka, 2016). To date, however, only *P. penetrans* and *P. hippeastri* have been reported in strawberry farms in Costa Rica (Brenes-Campos et al., 2021; Sandoval-Ruiz et al., 2023).

*Meloidogyne* and *Pratylenchus* were found coexisting in approximately one-third of the root samples in this survey. Producers and agricultural extensionists should keep this in mind to avoid further dissemination of these nematodes through contaminated material, and for agronomic practices. Strawberry farmers, especially those who rotate strawberries with vegetables such as lettuce, carrots, potatoes, tomatoes, celery and others, should be aware that these crops are also hosts of *M. hapla* and *P. penetrans* (López and Salas, 1978; Fernández-Solano and Quesada-Solís, 2013; Desaeger, 2018; Sandoval et al., 2023).

In our study, multiple taxa of PPNs in strawberry plantations are common. However, the effects of possible cumulative nematode damage on strawberry growth and yield are unknown. Studies have suggested that yield losses and increased susceptibility to other soil-borne pathogens are associated with *M. hapla* and *P. penetrans,* respectively (LaMondia, 1999; Desaeger, 2018).

In Costa Rica, importing strawberry planting material from other countries is a common practice (MAG, 2017). When not properly monitored, this can have unintended consequences, such as allowing the entry of nematodes into new areas (Nyoike et al., 2012). Although *A. fragariae* and *A. besseyi* have not yet been detected affecting strawberry farms in the country, they pose a potential threat to national production not only of strawberry, but of ornamental crops as well (Watson et al., 2020; Subbotin, 2024), especially when most of the planting material is imported.

In Costa Rica, there has been a limited amount of research focused on the study of the diversity of PPNs for berry crops such as strawberry, goldenberry, blueberry, and blackberry — the main berries produced in the Central Region. To our knowledge, only one survey has been conducted in blackberry (Peraza-Padilla and Orozco-Aceves, 2018). Currently, the use of broad-spectrum chemicals such as carbamates and organophosphates has been restricted or prohibited due to their impact on the environment and human health (Jones, 2017). Management of PPNs has been focused on the integration of practices that allow more efficient, prolonged, and selective control (Bucki et al., 2020; Desaeger et al., 2020). The correct diagnosis of PPNs is valuable for informing more specific actions for agronomic management. Future efforts should be focused on determining the population densities of PPNs in the main strawberry cultivars used in Costa Rica, particularly their impact on yield and possible interactions with other soil pathogens.
